# Assessing future heat stress across China: combined effects of heat and relative humidity on mortality

**DOI:** 10.3389/fpubh.2023.1282497

**Published:** 2023-10-03

**Authors:** Guwei Zhang, Ling Han, Jiajun Yao, Jiaxi Yang, Zhiqi Xu, Xiuhua Cai, Jin Huang, Lin Pei

**Affiliations:** ^1^Institute of Urban Meteorology, China Meteorological Administration, Beijing, China; ^2^Key Laboratory of Urban Meteorology, China Meteorological Administration, Beijing, China; ^3^Key Laboratory of Transforming Climate Resources to Economy, China Meteorological Administration, Chongqing, China; ^4^National Key Laboratory of Intelligent Tracking and Forecasting for Infectious Diseases, National Institute for Communicable Disease Control and Prevention, Chinese Center for Disease Control and Prevention, Beijing, China; ^5^Shengzhou Meteorological Bureau, Shaoxing, China; ^6^Chinese Academy of Meteorological Sciences, Beijing, China; ^7^Chifeng City Center Hospital Ningcheng County, Chifeng, China

**Keywords:** NEX-GDDP-CMIP6, China, heat stress, heat-related mortality, future projections

## Abstract

This study utilizes China’s records of non-accidental mortality along with twenty-five simulations from the NASA Earth Exchange Global Daily Downscaled Projections to evaluate forthcoming heat stress and heat-related mortality across China across four distinct scenarios (SSP1-2.6, SSP2-4.5, SSP3-7.0, and SSP5-8.5). The findings demonstrate a projected escalation in the heat stress index (HSI) throughout China from 2031 to 2100. The most substantial increments compared to the baseline (1995–2014) are observed under SSP5-8.5, indicating a rise of 7.96°C by the year 2100, while under SSP1-2.6, the increase is relatively modest at 1.54°C. Disparities in HSI growth are evident among different subregions, with South China encountering the most significant elevation, whereas Northwest China exhibits the lowest increment. Projected future temperatures align closely with HSI patterns, while relative humidity is anticipated to decrease across the majority of areas. The study’s projections indicate that China’s heat-related mortality is poised to surpass present levels over the forthcoming decades, spanning a range from 215% to 380% from 2031 to 2100. Notably, higher emission scenarios correspond to heightened heat-related mortality. Additionally, the investigation delves into the respective contributions of humidity and temperature to shifts in heat-related mortality. At present, humidity exerts a greater impact on fluctuations in heat-related mortality within China and its subregions. However, with the projected increase in emissions and global warming, temperature is expected to assume a dominant role in shaping these outcomes. In summary, this study underscores the anticipated escalation of heat stress and heat-related mortality across China in the future. It highlights the imperative of emission reduction as a means to mitigate these risks and underscores the variances in susceptibility to heat stress across different regions.

## 1. Introduction

In the past decade (2011–2020), carbon emissions have reached unprecedented levels in human history, coinciding with a surge in occurrences of extreme heat events globally (1, 2). The rapid pace of global warming has propelled heat stress to the forefront as a highly perilous climate risk, impacting public health, socioeconomics, and the ecological environment (3). Elevated ambient temperatures can elevate the body’s core temperature and heart rate, leading to conditions like heatstroke, respiratory and circulatory disorders, and even fatalities (4).

Noteworthy past events serve as stark reminders of the dangers associated with extreme heat. For instance, the 1995 heatwave in Chicago, USA claimed the lives of over 700 individuals (5). The record-breaking European heatwaves of 2003 resulted in substantial loss of life and economic devastation (6). Similarly, the 2010 heat event in Russia led to more than 50,000 fatalities (7). More recent occurrences include the North American super-heatwave in 2021, which claimed the lives of over 500 people (8). China has also faced significant negative impacts due to extreme heat events (9–13). According to Cai et al. (9, 10), nearly 15,000 deaths in China during 2020 were attributed to heat events. The escalating impact of climate change underscores the impending severity of heat stress, particularly in densely populated regions around the world (14–16). Given the pressing nature of the current scenario, it is of paramount importance to conduct a thorough assessment of future heat-related risks and undertake comprehensive measures to combat climate change, including emission reduction and strategic planning (1, 2).

Global climate models represent a cutting-edge tool for understanding climate change and projecting future scenarios. The recent Intergovernmental Panel on Climate Change (IPCC) report integrates various factors to quantify development and climate change (17). This integration involves combining shared socioeconomic pathways (SSPs) with representative concentration pathways (18, 19). In line with these novel scenarios, the IPCC introduced the Coupled Model Intercomparison Project Phase 6 (CMIP6), which furnishes the latest projections for future climate change (20).

While extensive efforts have been invested in leveraging the CMIP6 dataset to project future climate (16, 21, 22), the inherent coarse horizontal resolution (ranging from 1° to 3°) of the raw CMIP6 models poses challenges for in-depth regional climate analysis and introduces uncertainties due to model biases. The low resolution of CMIP6 poses a challenge in accurately simulating the impacts of urbanization on local climate. This limitation is particularly problematic for future public health risk assessments, given the substantial urban population (11, 16). Additionally, research has revealed that the original CMIP6 models tend to overestimate future temperatures, with overestimations ranging from 3.4% to 11.6% (23). To address these limitations, NASA has released the NASA Earth Exchange Global Daily Downscaled Projections (NEX-GDDP-CMIP6) dataset (24). These downscaled products are derived from the Bias Correction Spatial Disaggregation (BCSD) method, producing daily variants with an enhanced horizontal resolution of 0.25°. This refinement enhances both simulation accuracy and spatial resolution compared to the original CMIP6 outputs (25, 26). Previous research has demonstrated the alignment of NEX-GDDP-CMIP6 with observational data for modeling daily metrics (27, 28). Wu et al. (28) validated the capability of NEX-GDDP-CMIP6 to replicate China’s spatial temperature characteristics. Nonetheless, existing studies employing NEX-GDDP-CMIP6 have predominantly focused on predicting future extreme climate changes, often lacking comprehensive risk assessments (28–30). The NEX-GDDP-CMIP6 dataset provides valuable information for climate change research, impact assessments, and adaptation planning. Understand and respond better to the potential risks as well as impacts of future climate change. To provide better information for governmental strategic planning, it is necessary to employ this dataset to project future heat health risks in China.

Accordingly, this study plans to utilize the newly released and updated high-resolution NEX-GDDP-CMIP6 to project future changes in heat stress across China and its seven sub-regions under various emission scenarios with the heat stress index (HSI) that take into account both air temperature and humidity. Further, we will also employ the HSI-mortality exposure-response relationship and future population datasets to assess future mortality changes and quantify the contributions from temperature and humidity to heat mortality changes. This study aims to project future risks from multiple perspectives to support climate mitigation and strategic planning.

## 2. Data and methods

### 2.1. Historical mortality records

The Chinese Centre for Disease Control and Prevention (CDC) furnishes daily records of non-accidental death occurrences. Following the guidelines of the International Classification of Diseases-10th Revision (ICD-10), non-accidental deaths pertain to fatalities resulting from diseases rather than injuries. In the scope of this study, a total of 195 surveillance sites capturing mortality were encompassed (Supplementary Table S1). Recorded fatalities were observed across the majority of districts spanning the years 2010 to 2016. However, in the case of Guangzhou and Xining, recorded deaths were limited to the timeframe of 2012 to 2016.

### 2.2. Climate data

With a spatial resolution of 0.25° × 0.25°, the NEX-GDDP-CMIP6 dataset (24) offers a collection of scientifically downscaled climatic scenarios spanning from 1950 to 2100. This dataset utilizes the BCSD algorithm in combination with observational data generation to perform bias correction and downscaling of the CMIP6 model outputs. The BCSD method represents a trend-sustaining statistical downscaling technique that has gained widespread use in meteorology (25, 26, 31). The variational approach employed involves comparing the output from global climate models with real-world climate observations from a common reference period. This information is then employed to modify future climate projections to enhance their congruence with historical records and to enhance the accuracy of specific spatial regions. Leveraging the spatial granularity of the observed dataset, the algorithm additionally interpolates the output from global climate models onto a more refined grid, thereby enhancing spatial resolution.

We employed a total of twenty-five models sourced from the NEX-GDDP-CMIP6 dataset (Supplementary Table S2), selected based on their availability of daily mean relative humidity and surface air temperature. This set of models encompasses historical simulations spanning the period from 1995 to 2014, as well as future projections from 2015 to 2100. These projections are offered across four distinct emission scenarios: SSP1-2.6, SSP2-4.5, SSP3-7.0, and SSP5-8.5. These scenarios span a spectrum of carbon emissions, ranging from high to low levels. The four chosen scenarios are considered Tier 1 scenarios and are mandatory for all climate models participating in the Scenario Model Intercomparison Project (SMIP) of CMIP6. In essence, they represent the four emission trajectories that current climate research indicates are the most probable paths for the world to take in the future. Specifically, among these scenarios, SSP5-8.5 is the sole scenario that yields a radiative forcing of 8.5 W·m^−2^ in the year 2100, indicating a high level of emissions. SSP3-7.0 combines a relatively elevated degree of social vulnerability with a forcing of 7.0 W·m^−2^. In the context of SSP2-4.5, a moderate level of social vulnerability aligns with a moderate forcing level of 4.5 W·m^−2^. SSP1-2.6, on the other hand, combines attributes of low vulnerability, limited mitigation challenges, and a low forcing level of 2.6 W·m^−2^.

### 2.3. Population data

Under the umbrella of the four SSPs—namely, SSP1, SSP2, SSP3, and SSP5—the population dataset offers global population estimates spanning intervals of a decade from 2010 to 2100, with a spatial resolution of 0.125° (32). Each SSP corresponds to a distinct developmental trajectory: SSP1 signifies sustainable development characterized by reduced reliance on natural and fossil fuels. SSP2 embodies a business-as-usual scenario that maintains the growth patterns of recent decades, achieves growth targets, and progressively diminishes reliance on fossil fuels. SSP3 encapsulates a global landscape of regional competition, featuring pronounced disparities between regions, a significant gap between affluence and poverty, challenges in achieving developmental objectives, and escalating reliance on fossil fuels. SSP5 represents a fossil-fueled development approach, prioritizing economic expansion and addressing socioeconomic issues through self-interested actions (19). To ensure compatibility with the NEX-GDDP-CMIP6, we performed bilinear interpolation to adjust the population data to a resolution of 0.25° × 0.25°.

### 2.4. Study periods and regions

Consistent with previous studies (16, 33), the period from 1995 to 2014 is designated as the baseline, while the period spanning 2030 to 2100 is considered as the future timeframe. Additionally, we have segmented the future into 10 years intervals to analyze the projections for each decade: the 2030s (2030–2039), 2040s (2040–2049), 2050s (2050–2059), 2060s (2060–2069), 2070s (2070–2079), 2080s (2080–2089), and 2090s (2090–2099).

**Figure 1:**
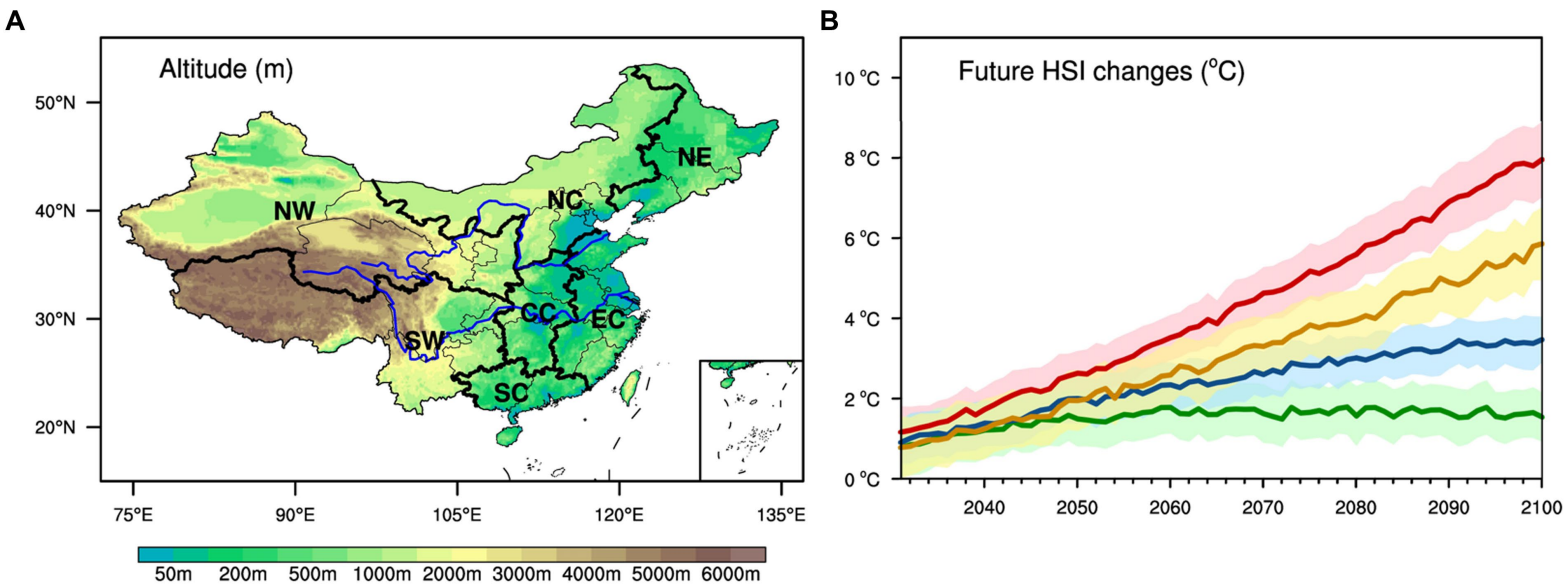
**(A)** Cartographic representation of China’s topography (units: meters). **(B)** Changes in China’s annual HSI projected for the period 2031–2100, relative to the present levels (units: °C). The delineations in green, blue, yellow, and red correspond to SSP1-2.6, SSP2-4.5, SSP3-7.0, and SSP5-8.5, respectively. The shaded area depicts the model’s variations within a 95% CI.

The geographic zoning of China (Figure 1A) is used to identify seven subregions: South China (SC), East China (EC), Northeast China (NE), Northwest China (NW), North China (NC), Central China (CC), and Southwest China (SW).

### 2.5. Calculation for heat stress index

Episodic temperature is a measurement of heat stress in humans that takes into account ambient factors such as temperature and humidity (34, 35). The HSI is a composite index that combines temperature and humidity to establish an equivalent temperature that reflects human perception (36). It is derived from Rothfusz’s multiple regression analysis and is as follows.


(1) $$\begin{array}{c} \mathrm{HSI}=2.04901523\times T-42.379+10.14333127\times \mathrm{RH}\\ -\;0.22475541\times T\times \mathrm{RH}-0.00683783\times T^{2}\\ -\;0.05481717\times {\mathrm{RH}}^{2}+0.00122874\times \mathrm{RH}\times T^{2}\\ +\;0.00085282\times T\times {\mathrm{RH}}^{2}-0.00000199\times T^{2}\times {\mathrm{RH}}^{2} \end{array}$$

If the relative humidity drops below 13% and the temperature falls within the range of 26.7°C to 44.5°C, subtract the specified value, Adj, from the HSI, where *T* represents the temperature in degrees Celsius, HSI signifies the heat stress in degrees Celsius, and RH represents the relative humidity in percent.


(2) $$\mathrm{Adj}=\frac{13-\mathrm{RH}}{4}\times \sqrt{1-\frac{\left|9T-315\right|}{85}}$$

For relative humidity above 85% and temperatures between 26.7°C and 30.5°C, add the following Adj to the HSI:


(3) $$\mathrm{Adj}=\frac{\mathrm{RH}-85}{10}\times \frac{275-9T}{25}$$

In situations where temperature and relative humidity outcomes indicate that the HSI value falls below approximately 26.7°C, the applicability of the Rothfuss regression method becomes limited. In such cases, a more straightforward formula can be employed to compute values that align with the results derived from Steadman’s approach (35).


(4) HSI=1.98T+24.9+0.047RH

### 2.6. HSI-mortality relationship

Utilizing data from 195 sites in China, a two-stage analysis is employed to quantify the existing relationship between the HSI and mortality. In the initial stage, daily recorded data on HSI and mortality are employed to create quasi-Poisson regressions integrated with distributed lag nonlinear models (DLNM). This approach is adopted to establish the connection between HSI and mortality for each specific location, following the methodology outlined by Gasparrini et al. (37). Within the DLNM framework, cross-basis functions are introduced to model the non-linear and lagged effects of HSI on mortality. To facilitate prediction and compute the reference prediction, the “crosspred” function is employed.


(5) $$\log\left[E\left({\mathrm{mort}}_{i}\right)\right]=\alpha +\beta {\mathrm{HSI}}_{i,l}+ns\left(\mathrm{time,df}\right)+\mathrm{DOW}$$

where mort*_i_* is the daily mortality on the day *i*. The parameter *α* stands for the intercept. DOW signifies the impact of the day of the week. HSI_i,l_ denotes the cross-base matrix of the two dimensions of the HSI and lag days. *β* is the coefficient vector for HSI_i,l_. *ns* is the normal three-spline function. df represents the degree of freedom. For controlling long-term trends, a natural cubic spline with 7 degrees of freedom per year is employed for a time. Our previous studies have indicated that heat has an impact on mortality within a period of approximately 3 weeks, taking into consideration potential influences related to harvesting (38). Therefore, a lag of 21 days is selected, which is considered adequate for capturing the hysteresis effect of temperature without excessive complexity. The association between cumulative temperature and mortality in each district or county is quantified as a relative risk. The risk for each temperature series is compared to the minimum mortality HSI, which represents the HSI with the lowest mortality risk. Additional insights can be derived from prior studies (14, 38).

Owing to variations in health impacts between urban and rural settings, it was not feasible to assess the HSI-mortality relationship within provincial capitals. However, it’s noteworthy that heat-related risks exhibit similarities within the same climatic subregions (39). In the subsequent phase, a multivariate meta-analysis is conducted using the constrained maximum likelihood approach to investigate the HSI-mortality relationship. This analysis aims to reveal zonal patterns in the risk of HSI-related mortality. Subsequently, the best linear unbiased prediction (BLUP) method is employed to forecast the cumulative HSI-mortality relationship for each subregion. Detailed procedures can be found in the work by Gasparrini et al. (40). Heterogeneity is assessed through the utilization of Cochran’s *Q* approach and an extension of the *I*^2^ statistic.

The methodology described above for analyzing the relationship between HSI and mortality is similar to the widely accepted approach used for analyzing the relationship between temperature and mortality. By aggregating data points from a specific region, it is possible to construct an HSI-mortality exposure-response curve that provides an overall understanding of the impact within that region (15, 41, 42).

### 2.7. Estimated heat-related mortality

Following a methodology similar to the prior investigations (14, 38, 43, 44), we conduct estimations of heat-related mortality utilizing the NEX-GDDP-CMIP6 datasets. The number of daily HSI-related deaths for each grid point is first calculated. Deaths due to high temperatures are calculated by summing the subset of days with temperatures above minimum mortality HSI (relative risk of 1). Then we sum the deaths with HSI above the minimum mortality HSI to give the number of heat-related deaths for the year. Additionally, the heat-related mortality is derived by dividing the number of heat-related deaths by the relevant grid population. Importantly, it’s noteworthy that regional heat-related mortality pertains to the ratio of regional heat-related deaths to the regional population. The calculation methodology is outlined as follows:


(6) $$\mathrm{AF}=\frac{\mathrm{RR}-1}{\mathrm{RR}}$$

(7) $$D_{x,d}=Mt_{x,y}\times P_{x,y}\times {\mathrm{AF}}_{x,d}$$

(8) $${\mathrm{HD}}_{x,y}=\sum \left\{D_{x,d}\right\}\ldots \ldots \left(\mathrm{When}\;{\mathrm{HSI}}_{x,d}>\mathrm{MMHSI}\right)$$

(9) $${\mathrm{HM}}_{x,y}=\frac{{\mathrm{HD}}_{x,y}}{{\mathrm{Pop}}_{x,y}}$$

(10) $$\mathrm{Reg}\_{\mathrm{HD}}_{y}=\sum \left\{{\mathrm{HD}}_{x,y}\right\}$$

(11) $$\mathrm{Reg}\_{\mathrm{HM}}_{y}=\frac{\mathrm{Regional}\_{\mathrm{HD}}_{y}}{\sum \left\{{\mathrm{Pop}}_{x,y}\right\}}$$

**Figure 2:**
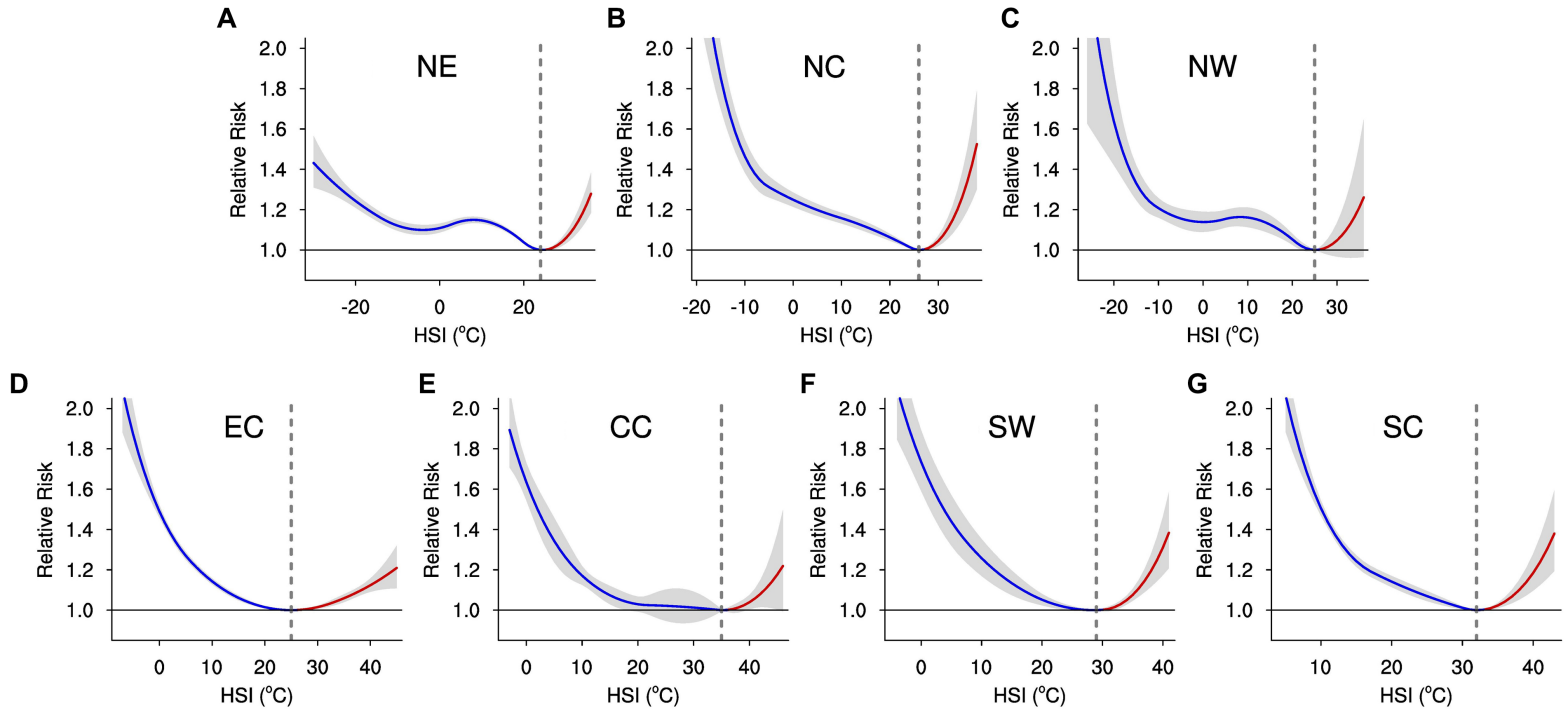
Collective cumulative non-linear associations between the HSI and daily mortality across lag days ranging from 0 to 21 are presented for each subregion within China. The curved lines illustrate the relative risk of HSI in comparison to the optimal HSI (signified by vertical gray dashed lines) associated with the lowest risk. The red and blue curved lines indicate the impact of heat and cold, respectively. The shaded regions encompass the 95% CI. **(A–G)** are for NE, NC, NW, EC, CC, SW, and SC, respectively.

The attribute fraction (AF) for a specific HSI value is computed from the relative risk (RR) determined using DLNM and meta-analyses across distinct subregions. This calculation assumes a consistent exposure-response relationship throughout the study timeframe. In the provided equations, *P*_x,y_ and *Mt*_x,y_ are the population and baseline mortality at grid *x* in year *y*. *D*_x,d_ and HSI_x,d_ are the daily HSI-related deaths and the HSI for day *d* in year *y*. HM_x,y_ (Reg_HM*_y_*) and HD_x,y_ (Reg_HD*_y_*) symbolize the (regional) annual heat-related mortality and deaths, respectively. In this study, the minimum mortality HSI for NE, NC, NW, EC, CC, SW, and SC is about 24°C, 26°C, 25°C, 25°C, 35°C, 29°C, and 32°C, respectively (Figure 2). The above steps are performed for each model and then average the outcomes of the models to get the ensemble mean.

### 2.8. Uncertainty analysis

Similar to previous research (21, 45), the principal sources of uncertainty in projecting future heat-related mortality within this study are attributed to the relationship between the HSI and mortality, as well as the divergences in HSI emanating from different model simulations. To address these uncertainties, a methodology involving Monte Carlo simulation (46) is harnessed to generate 1,000 samples of adjusted BLUP coefficients. This approach operates on the assumption that the estimations adhere to a multivariate normal distribution.

Subsequently, evaluations are conducted for each selected NEX-GDDP-CMIP6 simulation (45, 47). By aggregating the outputs from NEX-GDDP-CMIP6, the ensemble mean across multiple models is adopted as a representative depiction of the overall outcomes (16). The associated level of uncertainty is conveyed using a 95% confidence interval (CI). This interval, spanning the spectrum from the 2.5th to the 97.5th percentiles of the empirical distribution across the NEX-GDDP-CMIP6 results, serves as a quantification of the extent of uncertainty.

### 2.9. Contributions attributed to temperature and humidity

To examine the effects of temperature and humidity variations on heat-related mortality, we employ the Gini importance metric derived from the Random Forest algorithm (48). This approach allows us to elucidate the separate contributions of these intrinsic factors. The architecture of the random forest consists of decision trees, each comprising internal nodes and leaves. These internal nodes use specific features to split the dataset into two subsets with similar outcomes. Feature selection criteria, such as Gini impurity for classification or information gain, along with variance reduction for regression, guide the choice of features at internal nodes. The reduction in impurity attributed to each feature is measured, and the feature that leads to the most significant reduction is selected as the internal node. The significance of a feature is determined by calculating the average reduction in impurity across all trees in the forest.

In this study, we evaluate the individual impacts of temperature and humidity on heat-related mortality in each subregion across different scenarios. Utilizing data from multiple models, we incorporate annual temperature, humidity, and heat-related mortality data into the Random Forest model for simulation at each inhabited grid point within the subregion. It’s important to note that we exclusively consider temperature and humidity values for days when the HSI exceeds the minimum mortality HSI. The resulting Gini importance values for temperature and humidity, obtained from the Random Forest analysis, are scaled by a factor of 100% to represent their respective distinct contributions.

## 3. Results

### 3.1. Future heat stress changes across China

The future annual HSI for China is anticipated to escalate across various scenarios, with the most substantial increase observed under SSP5-8.5 (Figure 1B). Specifically, for SSP5-8.5, the projected HSI increase in 2031 is estimated to be 1.16°C (95% CI: 0.51–1.81°C) higher than the present, and this elevation is anticipated to reach 7.96°C (95% CI: 7.02–8.90°C) by the year 2100. In contrast, under lower emission scenarios such as SSP1-2.6, the projected HSI elevations in China are forecasted to be 0.78°C (95% CI: 0.06–1.54°C) in 2031 and merely 1.54°C (95% CI: 0.92–2.16°C) by 2100. This projection under SSP1-2.6 represents approximately 20% of the increase witnessed under SSP5-8.5.

**Figure 3:**
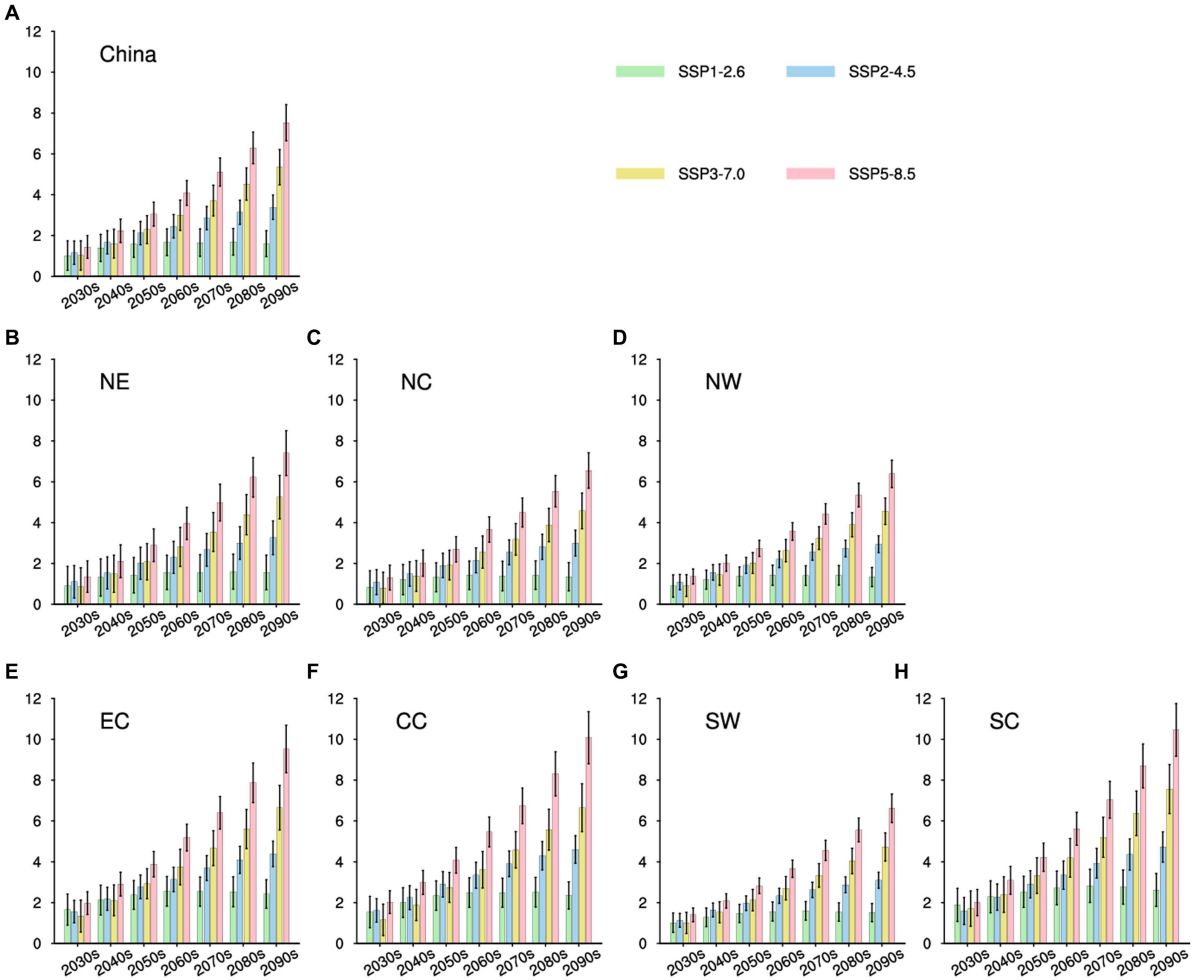
Projected alterations in HSI for China and its seven subregions are illustrated. The bars in green, blue, yellow, and red symbolize the averaged results for every 10 years under SSP1-2.6, SSP2-4.5, SSP3-7.0, and SSP5-8.5, respectively (units: °C). At the upper part of each bar, a vertical black line signifies the range of model variations within a 95% confidence interval. **(A–H)** are for China, NE, NC, NW, EC, CC, SW, and SC, respectively.

Importantly, it is noteworthy that under the SSP1-2.6 scenario, the growth of HSI will reach a plateau after 2060 and exhibit a decline in the 2090s (Figure 3A). Specifically, during the 2090s, China is projected to encounter an annual HSI increase of about 1.60°C (95% CI: 0.96–2.23°C), which is lower than the increase of 1.67–1.69°C (95% CI: 0.99–2.34°C) anticipated during the period from the 2060s to the 2080s. This disparity could be attributed to the fact that under SSP1-2.6, China’s temperature escalation will plateau by the end of the century, while humidity levels are projected to decrease (Supplementary Figures S1A, S2A). In contrast, under other scenarios (SSP2-4.5, SSP3-7.0, and SSP5-8.5), despite a projected decline in humidity after the 2060s (Supplementary Figure S2A), temperatures will persistently rise (Supplementary Figure S1A), thereby perpetuating the growth of HSI through the year 2100 (Figure 1B).

Focusing on future changes in the subregions, the projections consistently indicate significant HSI changes, with the highest increases observed under the high-emission scenario (Figure 3). Among the seven subregions, SC is projected to undergo the greatest HSI growth, with an estimated increase of 10.46°C (95% CI: 9.17–11.74°C) relative to the present in the 2090s under SSP5-8.5. Conversely, NW is projected to have the lowest increase among the subregions, approximately 6.39°C (95% CI: 5.72–7.05°C) under SSP5-8.5 in the 2090s. Furthermore, the HSI increases in NW, NC, NE, and SW are expected to be lower than the national average. Interestingly, these regions are all characterized by high latitude and high altitude areas in China.

The projections of future temperatures align closely with the HSI trends (Supplementary Figure S1), and there are regional differences in changes in relative humidity (Supplementary Figure S2). Overall, a decrease in relative humidity is anticipated for China and most regions in the future. However, the relative humidity in NC and NW is projected to increase across all scenarios, and additionally in EC and CC by 2070 under SSP3-7.0. When considering the future changes in HSI, temperature, and humidity together, although humidity is expected to decrease in most areas, the HSI remains relatively consistent with the temperature trend. This may be attributed to small variations in humidity (no more than ±3%), resulting in a minor effect on HSI.

### 3.2. Projected future HSI-related mortality

**Figure 4:**
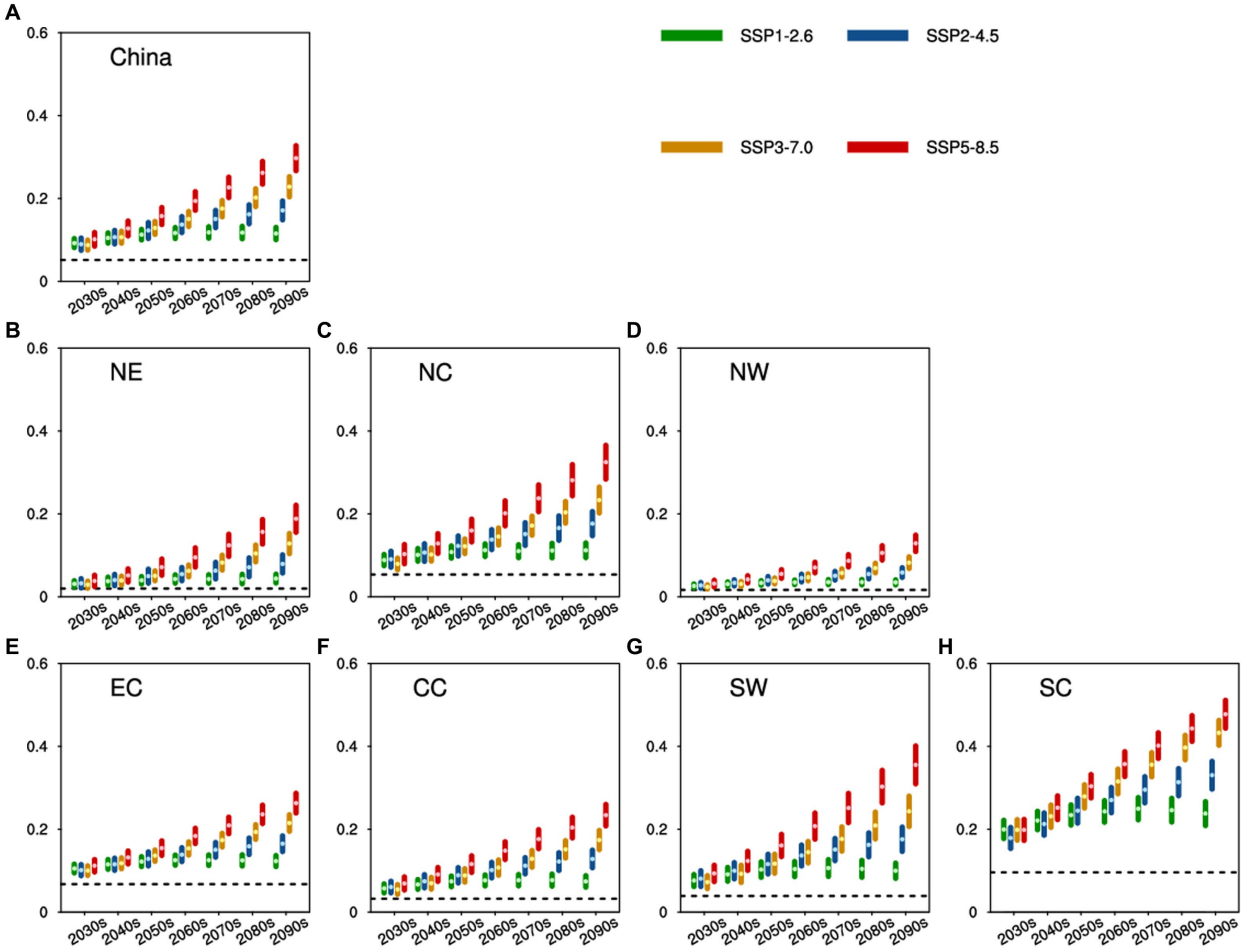
Future heat-related mortality for China and seven subregions. Green, blue, yellow, and red dots (accompanied by lines) signify the results (with model spreads shown within a 95% CI) averaged for every 10 years spanning 2031 to 2100 under SSP1-2.6, SSP2-4.5, SSP3-7.0, and SSP5-8.5, respectively (units: ‰). The black dashed lines depict the baseline heat-related mortality for each subregion. **(A–H)** are for China, NE, NC, NW, EC, CC, SW, and SC,respectively.

Drawing upon the historical correlations between HSI and mortality, as gleaned from the two-stage analysis (Figure 2), alongside the corresponding location-specific daily HSI data, we have derived estimations for the foundational heat-related mortality rates in China and its subregions (Supplementary Table S3). At present, SC exhibits the highest heat-related mortality, in contrast to NW which displays the lowest rates. Assuming a consistent relationship between HSI and mortality from the present into the future, we can project the heat-related mortality for the period between 2031 and 2100 by amalgamating the anticipated daily HSI values from NEX-GDDP-CMIP6 with the current exposure-response correlation (Figure 4).

In the forthcoming decades of the 21st century, heat-related mortality in China and its subregions is anticipated to exceed current levels across all scenarios. As depicted in the future HSI projections (Figure 3), heat-related mortality under high-emission scenarios will outpace that under low-emission scenarios. For instance, China’s annual heat-related mortality during the period 2031–2100 is projected to span from 0.09–0.12‰ (95% CI: 0.08–0.13‰), 0.09–0.17‰ (95% CI: 0.07–0.19‰), 0.09–0.23‰ (95% CI: 0.08–0.25‰), and 0.10–0.30‰ (95% CI: 0.08–0.33‰) for SSP1-2.6, SSP2-4.5, SSP3-7.0, and SSP5-8.5, respectively (Figure 4).

In contrast, scenarios that exclude SSP1-2.6 project a continued elevation in China’s heat-related mortality throughout the century, culminating in the highest rate during the 2090s. However, under the SSP1-2.6 scenario, heat-related mortality is expected to peak in the 2070s at around 0.12‰ (95% CI: 0.10–0.13‰). Consistent with regional variations in HSI, SC is anticipated to experience the highest heat-related mortality among the seven subregions, ranging approximately from 0.18–0.48‰ (95% CI: 0.15–0.51‰), while NW is projected to have the lowest, ranging approximately from 0.02–0.13‰ (95% CI: 0.02–0.15‰). Additionally, NC, SW, and CC are anticipated to have higher heat-related mortality compared to the national average in the future, whereas other subregions are predicted to remain below the national average.

**Figure 5:**
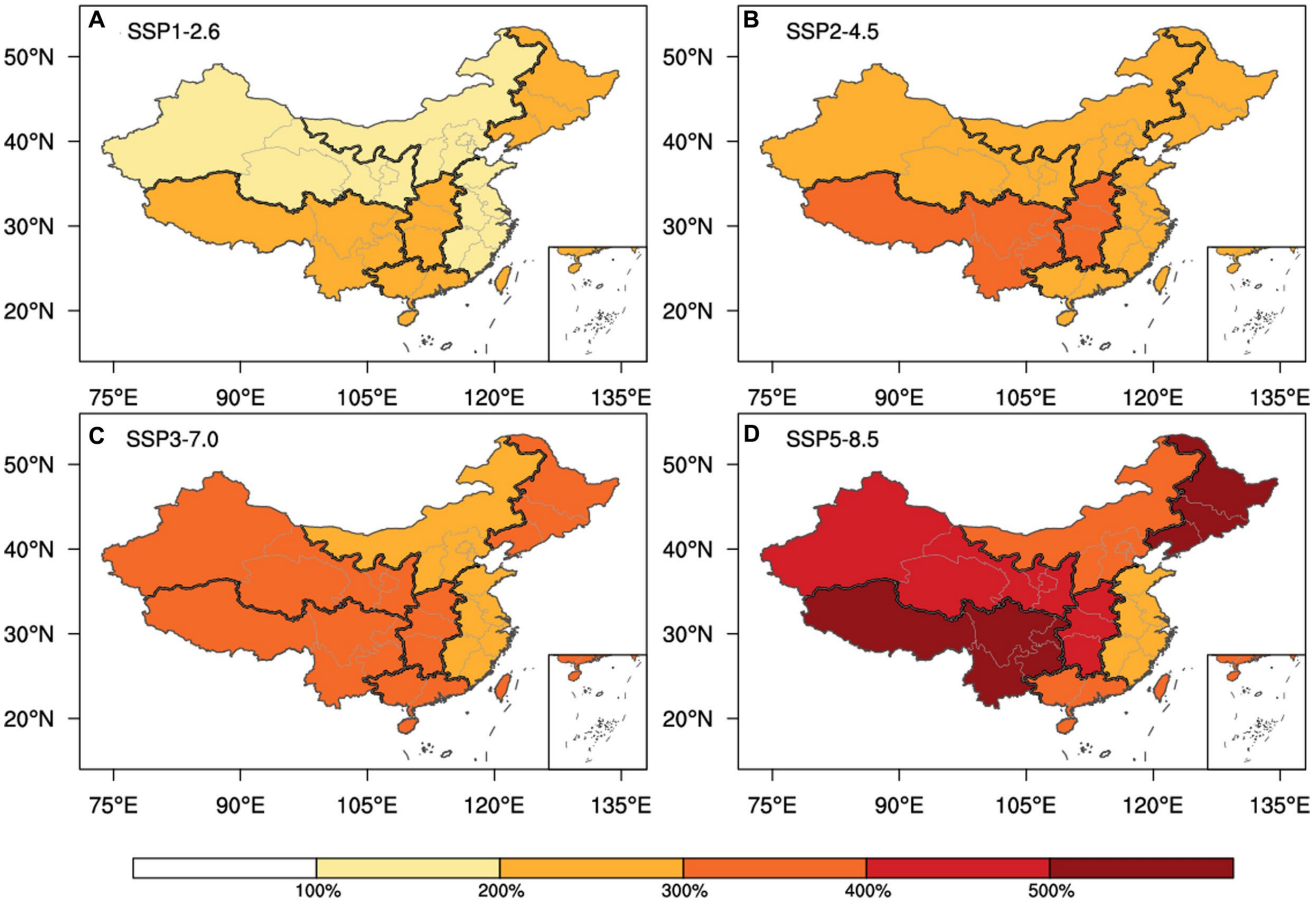
Altering proportions of upcoming heat-related mortality as compared to the present baseline, averaged across the period 2031 to 2100, across the scenarios SSP1-2.6 **(A)**, SSP2-4.5 **(B)**, SSP3-7.0 **(C)**, and SSP5-8.5 **(D)**.

Considering the anticipated alterations in future ratios as compared to the present (Figure 5), it is foreseeable that China’s annual heat-related mortality will undergo substantial escalation, encompassing a range of 215 to 380% overall. Notably, the most notable and minimal increments are projected under the SSP5-8.5 and SSP1-2.6 scenarios, respectively. This underscores the notion that even in the scenario where the most modest emission reduction goals established by the IPCC are realized, China’s heat-related mortality is still expected to double in comparison to the current baseline.

Among the various subregions, those situated at high latitudes and altitudes (excluding NC) are anticipated to undergo a more pronounced growth rate in heat-related mortality compared to other regions. Throughout 2031–2100, SW is expected to witness the highest annual increase in heat-related mortality, ranging approximately from 254% to 554% compared to the current level, marking the highest among the seven subregions. Conversely, EC is projected to experience the lowest increase, ranging approximately from 179% to 274%.

For all scenarios except SSP1-2.6, the average heat-related mortality in subregions during the period of 2031–2100 is projected to rise to at least 200% of their current levels. Notably, under SSP5-8.5, SW and NE will experience increases exceeding 500%. Analyzing the increases per decade (Supplementary Figure S3), differences among the scenarios are relatively modest in the 2030s, with almost no subregion surpassing a 200% increase. However, these differences become more pronounced in the subsequent decades. By the 2090s, heat-related mortality in most areas under SSP5-8.5 could be 6–7 times higher than the present levels, while under SSP1-2.6, it is projected to remain under 3 times the current level. This underscores the potential benefits of emission reduction in mitigating heat-related risks. However, it’s important to note that immediate success in risk reduction might not be achievable in the short term, emphasizing the necessity of strategic planning to effectively manage the impacts of climate change.

### 3.3. Temperature and humidity contributions to heat-related mortality

Assessing the individual impacts of temperature and humidity on heat vulnerabilities within each subregion can enhance the precision of climate change adaptation strategies (12, 42). Firstly, our analysis only considers grids with inhabited residences. Secondly, it’s important to clarify that we exclusively include dates linked to heat risk, where the HSI for a given day surpasses the minimum mortality HSI threshold (i.e., corresponding to heat-related mortality above 0). Consequently, our investigation focuses on understanding the impact of temperature or humidity on heat-related mortality when a state of vulnerability to heat has already been established. In simpler terms, the discussion regarding humidity’s influence on heat mortality does not suggest that humidity alone can create vulnerability to heat. Our consistent perspective is that the effect of humidity on the risk of heat-related mortality is contingent upon elevated temperature conditions.

**Figure 6:**
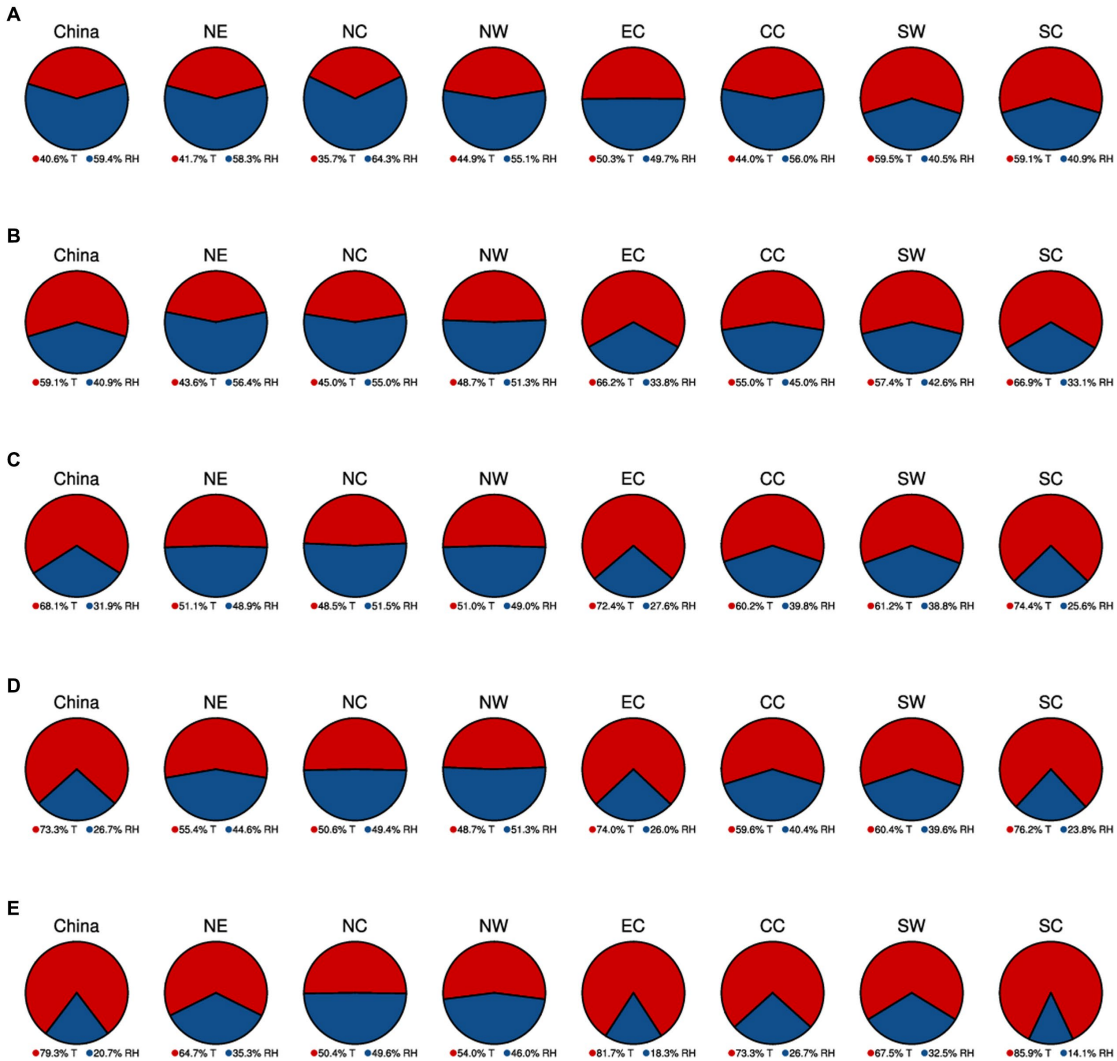
The pie charts illustrate the combined impacts of humidity (blue) and temperature changes (red) on heat-related mortality in China and its seven subregions for the period 2031 to 2100. These projections encompass scenarios from SSP1-2.6 **(A)**, SSP2-4.5 **(B)**, SSP3-7.0 **(C)**, and SSP5-8.5 **(D)**.

As depicted in Figure 6, the impacts of temperature and humidity on heat-related mortality in China and its subregions are analyzed for both current and future periods. Currently (Figure 6A), the contribution of China’s humidity and temperature to heat-related mortality change is about 59.4% and 40.6%, suggesting that humidity changes have a greater impact. Similar patterns are observed in NE, NC, NW, and CC, where humidity in NC contributes to 64.3% of heat-related mortality changes. In most southern areas (EC, SW, and SC), temperature plays a more important role.

In the future (2031–2100), with rising emissions and ongoing global warming, the influence of temperature will become more prominent (Figures 6B–E). For China, temperature is projected to be the predominant factor driving changes in heat-related mortality, contributing to 59.1%, 68.1%, 73.3%, and 79.3% of heat mortality changes under SSP1-2.6, SSP2-4.5, SSP3-7.0, and SSP5-8.5, respectively. Under SSP1-2.6 (Figure 6B), the humidity will still exert a stronger influence on heat mortality changes than the temperature in the northern subregions (NC, NE, and NW). However, its impact will diminish compared to the present, with the most significant decrease occurring in NC (almost 10%). Under SSP2-4.5 and SSP3-7.0 (Figures 6C,D), only one subregion each, NC and NW respectively, will have slightly higher contributions from humidity compared to temperature. In the case of SSP5-8.5, temperature will hold a greater sway on heat-related mortality than humidity across all subregions, particularly in SC, where temperature is projected to account for 85.9% of the contributions. Overall, the future impacts of temperature on heat-related mortality will amplify as emissions-driven climate warming intensifies.

## 4. Discussion

Humidity emerges as a pivotal factor in shaping heat-related health vulnerabilities. This study leverages the NEX-GDDP-CMIP6 datasets to project forthcoming heat-related mortality in China, incorporating the HSI that encapsulates both temperature and humidity. The results underscore a future surge in heat-related mortality across China and its subregions due to the confluence of climate change and emissions. A noteworthy and intriguing discovery lies in the higher contribution of humidity to changes in heat mortality within the northern subregions compared to their southern counterparts. This phenomenon might be attributed to the prevalence of wet and dry heat events, often coinciding with extreme heat occurrences. In regions marked by predominantly wet and warm conditions, like the south, high humidity during heat events mitigates the impact, rendering humidity-driven changes less influential. In contrast, the northern areas witness fluctuations in humidity that can convert a dry heat event into a moist one, leading the HSI to surpass critical risk thresholds. In such scenarios, the effect of humidity is amplified, accentuating its role in shaping heat-related mortality.

Furthermore, our findings reveal a noteworthy similarity in the anticipated heat-related hazards across various emissions scenarios prior to 2040. This resemblance can be attributed to the persistent nature of greenhouse gases in the environment, suggesting that alterations in carbon emissions will not yield immediate climate effects. Consequently, the populace of China will persist in confronting noteworthy heat vulnerabilities in the forthcoming decades. It’s important to emphasize that even within a trajectory of lower emissions (SSP1-2.6), the future toll of heat-related fatalities is projected to exceed current levels, thereby presenting substantial challenges for both emergency response protocols and healthcare systems. Hence, in conjunction with the endeavor to curtail emissions for the purpose of achieving carbon neutrality, it becomes imperative to formulate localized emergency strategies that adeptly manage the repercussions of extreme heat on human well-being.

Our findings exhibit both similarities and differences when compared to previous studies utilizing raw CMIP6 simulations (11, 12, 16, 33, 42, 49). Specifically, we find that future HSI with the related heat risks will increase with higher emissions across China, particularly in high latitude and high altitude areas. Additionally, we note that the humidity in NC is projected to increase in the future. This aligns with the findings of Zhang et al. (16), which indicated an increase in humidity for the Beijing-Tianjin-Hebei urban agglomeration. These consistent results support the robustness of our and previous conclusions. However, it is worth mentioning that the future increases in HSI projected in our study are slightly lower compared to previous CMIP6-based findings, particularly in northern areas. This discrepancy may be attributed to the bias correction and statistical downscaling applied by NEX-GDDP-CMIP6, which could lead to a reduction in the estimated future temperatures (24). Furthermore, our study suggests that future changes in humidity are unlikely to exceed ±3% across China, whereas a previous study indicated a reduction in humidity exceeding 4% in CC (16). The reasons behind these differences require further investigation and may also be associated with the BCSD method performed by NEX-GDDP-CMIP6.

This study presents several limitations that warrant consideration. The influence of societal progressions on human adaptation to elevated temperatures cannot be overlooked. Factors such as economic prosperity, medical advancements, and educational improvements can potentially reshape baseline mortality, consequently impacting heat-related fatalities. Within the scope of this investigation, we operate under the assumption that forthcoming populations will maintain a comparable baseline mortality rate and a similar relationship between HSI and mortality as observed presently. However, it’s important to acknowledge that the HSI-mortality relationship is poised to change in response to the evolution of human tolerance to elevated temperatures. For instance, as human resilience to heat stress strengthens, the HSI value associated with the lowest mortality is likely to rise. These dynamic elements introduce intricacies and uncertainties into the risk assessment undertaken within this study. Subsequent assessments could enhance precision in forecasting HSI-related fatalities by recalibrating exposure-response connections and baseline mortality figures, leveraging more advanced data. Additionally, it’s worth noting that the methodology of statistical downscaling primarily hinges on statistical correlations and pattern assimilation, without direct modeling of physical mechanisms. This could potentially introduce biases between downscaled projections and actual physical processes, particularly on a global scale where future conditions deviate from the present circumstances.

## 5. Conclusion

In summary, this study offers projections of forthcoming alterations in heat stress and heat-associated mortality across China and its subregions within the context of diverse emission scenarios. The outcomes underscore a universal escalation in the annual heat stress index (HSI) across all envisaged pathways, with the most significant surge anticipated within the high-emission context (SSP5-8.5). By the year 2100, China’s HSI will amplify by 7.96°C under SSP5-8.5. Notably, among the subregions, the most substantial HSI augmentation is expected within SC, whereas NW is poised to exhibit the slightest rise. Furthermore, the prognosis reveals a projected increase in future heat-related mortality, eclipsing current benchmarks across all scenarios. The acutest escalation in heat-related mortality emerges for SSP5-8.5. However, even within the relatively moderate emission framework of SSP1-2.6, China’s heat-related mortality is poised to potentially double the present rate. Within the analysis, we also delve into the contributions of temperature and humidity to shifts in heat-related mortality. Presently, humidity exerts a more pronounced influence on these variations. Yet, with the trajectory of heightened emissions and impending global warming, temperature is anticipated to evolve into the predominant determinant. These revelations underscore the exigency for adaptive strategies to ameliorate the repercussions of heat stress and heat-related mortality across China. The imperative lies in not only emissions reduction but also in the strategic implementation of planning measures to adeptly navigate climate transformations and safeguard public well-being.

## Code availability

The above analyses were performed using R (version 4.2), Python (version 3.9), and NCL (version 6.6), and the code is available on request.

## Data availability statement

The raw data supporting the conclusions of this article will be made available by the authors, without undue reservation.

## Author contributions

GZ: Conceptualization, Data curation, Funding acquisition, Methodology, Project administration, Writing – original draft, Writing – review & editing. LH: Data curation, Formal analysis, Methodology, Writing – review & editing. JnY: Investigation, Methodology, Project administration, Writing – review & editing. JiY: Conceptualization, Investigation, Project administration, Supervision, Validation, Visualization, Writing – review & editing, Funding acquisition, Resources. ZX: Data curation, Project administration, Supervision, Visualization, Writing – original draft. XC: Writing – review & editing. JH: Writing – review & editing. LP: Formal analysis, Investigation, Visualization, Writing – review & editing.

## References

[1] IPCC. Climate change 2021 In: Masson-Delmotte V Zhai P Pirani A Connors SL Péan C Berger S, editors. The physical science basis. Contribution of Working Group I to the sixth assessment report of the intergovernmental panel on climate change. Cambridge: Cambridge University Press (2021).

[2] IPCC. Climate change 2022: mitigation of climate change. *In:* PR Shukla R Slade Khourdajie AAl Van Diemen R D Mccollum M Pathak. Contribution of Working Group III to the sixth assessment report of the intergovernmental panel on climate change. Cambridge: Cambridge University Press (2022).

[3] Casanueva A Kotlarski S Herrera S Fischer AM Kjellstrom T Schwierz C. Climate projections of a multivariate heat stress index: the role of downscaling and bias correction. Geosci Model Dev. (2019) 12:3419–38. doi: 10.5194/gmd-12-3419-2019

[4] Baldwin JW Dessy JB Vecchi GA Oppenheimer M. Temporally compound heat wave events and global warming: an emerging hazard. Earths Future. (2019) 7:411–27. doi: 10.1029/2018EF000989

[5] Dematte JE O’Mara K Buescher J Whitney CG Forsythe S McNamee T. Near-fatal heat stroke during the 1995 heat wave in Chicago. Ann Intern Med. (1998) 129:173–81. doi: 10.7326/0003-4819-129-3-199808010-00001, PMID: 9696724

[6] Robine JM Cheung SL Le Roy S Van Oyen H Griffiths C Michel JP. Death toll exceeded 70,000 in Europe during the summer of 2003. C R Biol. (2008) 331:171–8. doi: 10.1016/j.crvi.2007.12.001, PMID: 18241810

[7] Fischer EM Knutti R. Anthropogenic contribution to global occurrence of heavy-precipitation and high-temperature extremes. Nat Clim Chang. (2015) 5:560–4. doi: 10.1038/nclimate2617

[8] Schiermeier Q. Climate change made North America’s deadly heatwave 150 times more likely. Nature. (2021). doi: 10.1038/d41586-021-01869-0, PMID: 34239114

[9] Cai W Zhang C Suen HP Ai S Bai Y Bao J. The 2020 China report of the Lancet Countdown on health and climate change. Lancet Public Health. (2021) 6:e64–81. doi: 10.1016/S2468-2667(20)30256-5, PMID: 33278345 PMC7966675

[10] Cai W Zhang C Zhang S Ai S Bai Y Bao J. The 2021 China report of the Lancet Countdown on health and climate change: seizing the window of opportunity. Lancet Public Health. (2021) 6:e932–47. doi: 10.1016/S2468-2667(21)00209-7, PMID: 34758286

[11] Yang J-X Zhou B-Q Zhai P-M. Constrained high-resolution projection of hot extremes in the Beijing–Tianjin–Hebei region of China. Adv Clim Chang Res. (2023) 14:387–93. doi: 10.1016/j.accre.2023.04.008

[12] Zhang G Ma J Meng C Wang J Xu Z Gou P. Increasing heatwave with associated population and GDP exposure in North China. Int J Climatol. (2023) 43:4716–32. doi: 10.1002/joc.8113

[13] Zhang GW Zeng G Yang XY Iyakaremye V. Two spatial types of North China heatwaves and their possible links to Barents-Kara Sea ice changes. Int J Climatol. (2022) 42:6876–89. doi: 10.1002/joc.7617

[14] Xing Q Sun Z Tao Y Zhang X Miao S Zheng C. Impacts of urbanization on the temperature-cardiovascular mortality relationship in Beijing, China. Environ Res. (2020) 191:110234. doi: 10.1016/j.envres.2020.110234, PMID: 32956657

[15] Yang J Zhou M Ren Z Li M Wang B Liu L. Projecting heat-related excess mortality under climate change scenarios in China. Nat Commun. (2021) 12:1039. doi: 10.1038/s41467-021-21305-1, PMID: 33589602 PMC7884743

[16] Zhang GW Zeng G Liang XZ Huang CR. Increasing heat risk in China’s urban agglomerations. Environ Res Lett. (2021) 16:064073. doi: 10.1088/1748-9326/ac046e

[17] Moss RH Edmonds JA Hibbard KA Manning MR Rose SK Van Vuuren DP. The next generation of scenarios for climate change research and assessment. Nature. (2010) 463:747–56. doi: 10.1038/nature08823 20148028

[18] Kriegler E O’Neill BC Hallegatte S Kram T Moss RH Lempert RJ. Socio-economic scenario development for climate change analysis In: CIRED working papers hal-00866437. Paris, France: CIRAD. (2010).

[19] O’Neill BC Kriegler E Ebi KL Kemp-Benedict E Riahi K Rothman DS. The roads ahead: narratives for shared socioeconomic pathways describing world futures in the 21st century. Glob Environ Change. (2017) 42:169–80. doi: 10.1016/j.gloenvcha.2015.01.004

[20] Eyring V Bony S Meehl GA Senior CA Stevens B Stouffer RJ. Overview of the Coupled Model Intercomparison Project Phase 6 (CMIP6) experimental design and organization. Geosci Model Dev. (2016) 9:1937–58. doi: 10.5194/gmd-9-1937-2016

[21] Wang J Chen Y Liao WL He GH Tett SFB Yan ZW. Anthropogenic emissions and urbanization increase risk of compound hot extremes in cities. Nat Clim Chang. (2021) 11:1084–9. doi: 10.1038/s41558-021-01196-2

[22] Wang J Chen Y Tett SFB Yan Z Zhai P Feng J. Anthropogenically-driven increases in the risks of summertime compound hot extremes. Nat Commun. (2020) 11:528. doi: 10.1038/s41467-019-14233-8, PMID: 32047147 PMC7012878

[23] Chai YF Yue Y Slater LJ Yin JB Borthwick AGL Chen TX. Constrained CMIP6 projections indicate less warming and a slower increase in water availability across Asia. Nat Commun. (2022) 13:424. doi: 10.1038/s41467-022-31782-7 35840591 PMC9287300

[24] Thrasher B Wang W Michaelis A Melton F Lee T Nemani R. Nasa global daily downscaled projections, CMIP6. Sci Data. (2022) 9:262. doi: 10.1038/s41597-022-01393-4, PMID: 35654862 PMC9163132

[25] Wood AW Leung LR Sridhar V Lettenmaier DP. Hydrologic implications of dynamical and statistical approaches to downscaling climate model outputs. Clim Chang. (2004) 62:189–216. doi: 10.1023/B:CLIM.0000013685.99609.9e

[26] Wood AW Maurer EP Kumar A Lettenmaier DP. Long-range experimental hydrologic forecasting for the eastern United States. J Geophys Res-Atmos. (2002) 107:ACL 6-1–ACL 6-15. doi: 10.1029/2001JD000659

[27] Chen M Chen L Zhou Y Hu M Jiang Y Huang D. Rising vulnerability of compound risk inequality to ageing and extreme heatwave exposure in global cities. npj Urban Sustain. (2023) 3:38. doi: 10.1038/s42949-023-00118-9

[28] Wu F Jiao D Yang X Cui Z Zhang H Wang Y. Evaluation of NEX-GDDP-CMIP6 in simulation performance and drought capture utility over China—based on DISO. Hydrol Res. (2023) 54:703–21. doi: 10.2166/nh.2023.140

[29] Xu L Zhang T Yu W Yang S. Changes in concurrent precipitation and temperature extremes over the Asian monsoon region: observation and projection. Environ Res Lett. (2023) 18:044021. doi: 10.1088/1748-9326/acbfd0

[30] Zhang Y You Q Ullah S Chen C Shen L Liu Z. Substantial increase in abrupt shifts between drought and flood events in China based on observations and model simulations. Sci Total Environ. (2023) 876:162822. doi: 10.1016/j.scitotenv.2023.162822, PMID: 36921874

[31] Thrasher B Maurer EP Mckellar C Duffy PB. Technical note: Bias correcting climate model simulated daily temperature extremes with quantile mapping. Hydrol Earth Syst Sci. (2012) 16:3309–14. doi: 10.5194/hess-16-3309-2012

[32] Jones B O’Neill BC. Spatially explicit global population scenarios consistent with the shared socioeconomic pathways. Environ Res Lett. (2016) 11:084003. doi: 10.1088/1748-9326/11/8/084003

[33] Zhang GW Zeng G Yang XY Jiang ZH. Future changes in extreme high temperature over China at 1.5 degrees C-5 degrees C global warming based on CMIP6 simulations. Adv Atmos Sci. (2021) 38:253–67. doi: 10.1007/s00376-020-0182-8

[34] Russo S Sillmann J Sterl A. Humid heat waves at different warming levels. Sci Rep. (2017) 7:7477. doi: 10.1038/s41598-017-07536-7, PMID: 28785096 PMC5547064

[35] Steadman RG. The assessment of sultriness. Part I: a temperature-humidity index based on human physiology and clothing science. J Appl Meteorol. (1979) 18:861–73. doi: 10.1175/1520-0450(1979)018<0861:TAOSPI>2.0.CO;2

[36] Buzan JR Oleson K Huber M. Implementation and comparison of a suite of heat stress metrics within the Community Land Model version 4.5. Geosci Model Dev. (2015) 8:151–70. doi: 10.5194/gmd-8-151-2015

[37] Gasparrini A Armstrong B Kenward MG. Distributed lag non-linear models. Stat Med. (2010) 29:2224–34. doi: 10.1002/sim.3940, PMID: 20812303 PMC2998707

[38] Xing Q Sun Z Tao Y Shang J Miao S Xiao C. Projections of future temperature-related cardiovascular mortality under climate change, urbanization and population aging in Beijing, China. Environ Int. (2022) 163:107231. doi: 10.1016/j.envint.2022.107231 35436720

[39] Chen H Zhao L Dong W Cheng L Cai W Yang J. Spatiotemporal variation of mortality burden attributable to heatwaves in China, 1979–2020. Sci Bull. (2022) 67:1340–4. doi: 10.1016/j.scib.2022.05.006, PMID: 36546266

[40] Gasparrini A Guo Y Hashizume M Lavigne E Zanobetti A Schwartz J. Mortality risk attributable to high and low ambient temperature: a multicountry observational study. Lancet. (2015) 386:369–75. doi: 10.1016/S0140-6736(14)62114-0, PMID: 26003380 PMC4521077

[41] Yang J Yin P Sun J Wang B Zhou M Li M. Heatwave and mortality in 31 major Chinese cities: definition, vulnerability and implications. Sci Total Environ. (2019) 649:695–702. doi: 10.1016/j.scitotenv.2018.08.332, PMID: 30176480

[42] Zhang G Sun Z Han L Iyakaremye V Xu Z Miao S. Avoidable heat-related mortality in China during the 21st century. npj Clim Atmos Sci. (2023) 6:81. doi: 10.1038/s41612-023-00404-4

[43] Hu J He G Meng R Gong W Ren Z Shi H. Temperature-related mortality in China from specific injury. Nat Commun. (2023) 14:37. doi: 10.1038/s41467-022-35462-4, PMID: 36596791 PMC9810693

[44] Huang C Cheng J Phung D Tawatsupa B Hu W Xu Z. Mortality burden attributable to heatwaves in Thailand: a systematic assessment incorporating evidence-based lag structure. Environ Int. (2018) 121:41–50. doi: 10.1016/j.envint.2018.08.058, PMID: 30172927

[45] Gasparrini A Guo Y Sera F Vicedo-Cabrera AM Huber V Tong S. Projections of temperature-related excess mortality under climate change scenarios. Lancet Planet Health. (2017) 1:360–367. doi: 10.1016/S2542-5196(17)30156-0, PMID: 29276803 PMC5729020

[46] Bonate PL. A brief introduction to Monte Carlo simulation. Clin Pharmacokinet. (2001) 40:15–22. doi: 10.2165/00003088-200140010-00002, PMID: 11236807

[47] Vicedo-Cabrera AM Sera F Gasparrini A. Hands-on tutorial on a modeling framework for projections of climate change impacts on health. Epidemiology. (2019) 30:321–9. doi: 10.1097/EDE.0000000000000982, PMID: 30829832 PMC6533172

[48] Breiman L. Random forests. Mach Learn. (2001) 45:5–32. doi: 10.1023/A:1010933404324

[49] Sun Z Wang Q Chen C Yang Y Yan M Du H. Projection of temperature-related excess mortality by integrating population adaptability under changing climate—China, 2050s and 2080s. China CDC Wkly. (2021) 3:697–701. doi: 10.46234/ccdcw2021.174, PMID: 34594971 PMC8422175

