# Supplementary material for: Assessing future heat stress across China: combined effects of heat and relative humidity on mortality
Source: Front Public Health. 2023 Oct 3;11:1282497. doi: 10.3389/fpubh.2023.1282497 (PMC10581210; doi:10.3389/fpubh.2023.1282497)
Supplement: Supplementary file 1 [file Data_Sheet_1.pdf]

frontiers

Supplementary Material

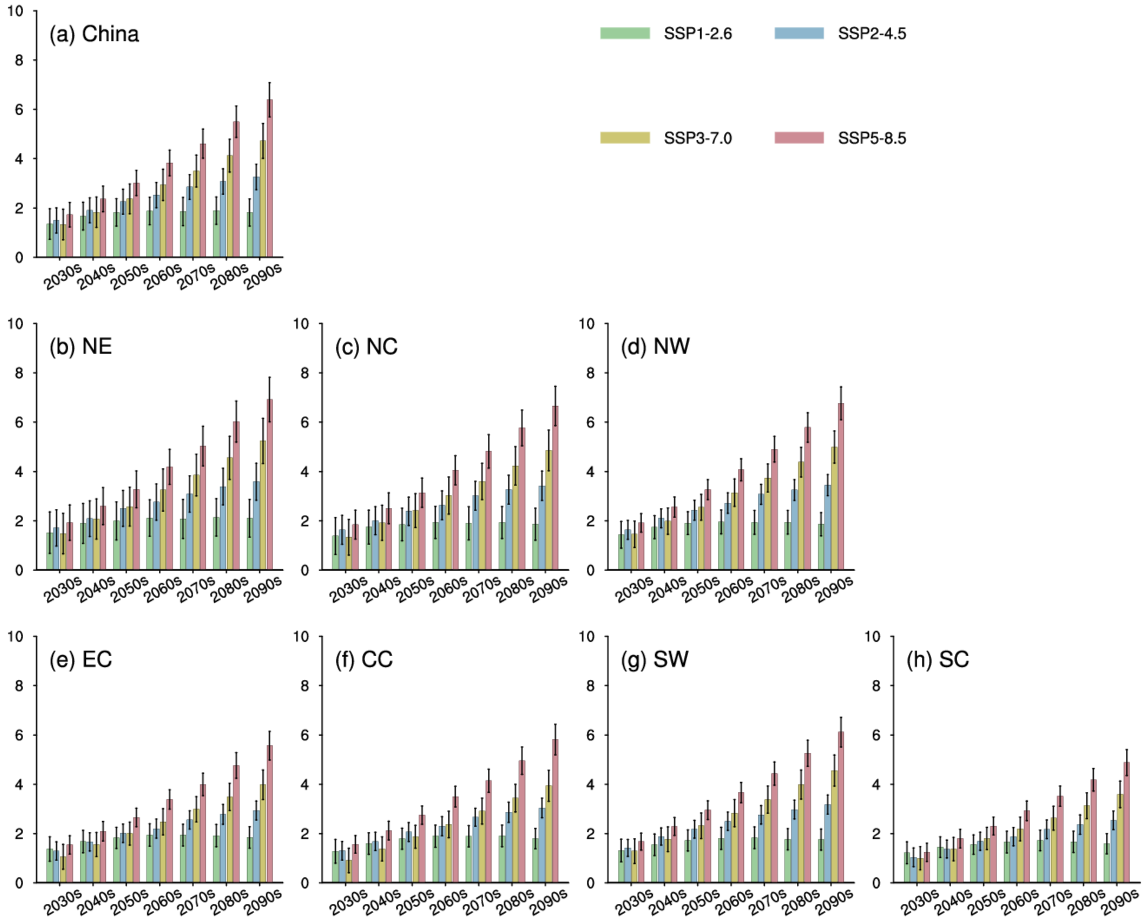

Supplementary Figure 1. Future temperature changes for China and seven subregions. Green, blue, yellow, and red bars indicate outcomes averaged for every 10-year interval under SSP1-2.6, SSP2-4.5, SSP3-7.0, and SSP5-8.5, respectively (units: °C). The vertical black line across the top of each bar depicts the model spreads with 95% CI.

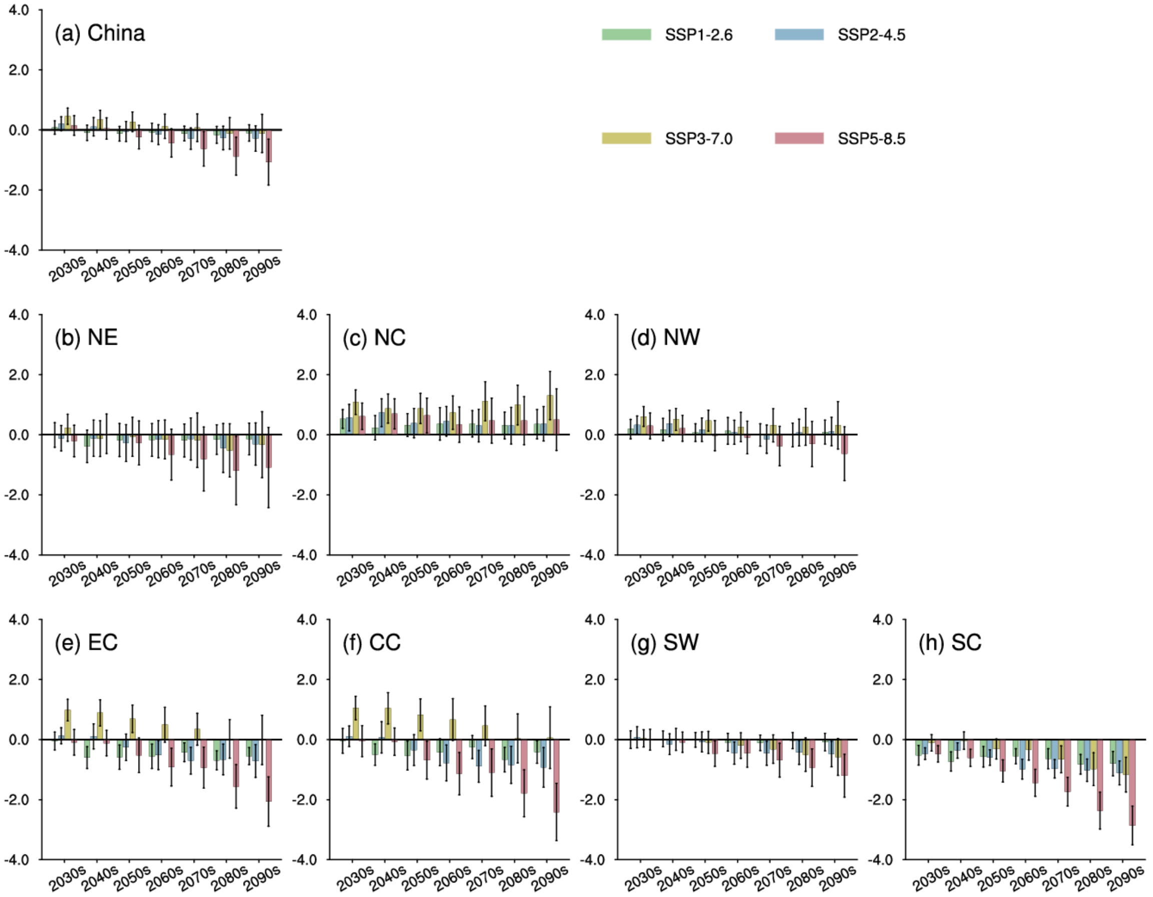

Supplementary Figure 2. Future relative humidity changes for China and seven subregions. Green, blue, yellow, and red bars indicate outcomes averaged for every 10-year interval under SSP1-2.6, SSP2-4.5, SSP3-7.0, and SSP5-8.5, respectively (units: %). The vertical black line across the top of each bar depicts the model spreads with 95% CI.

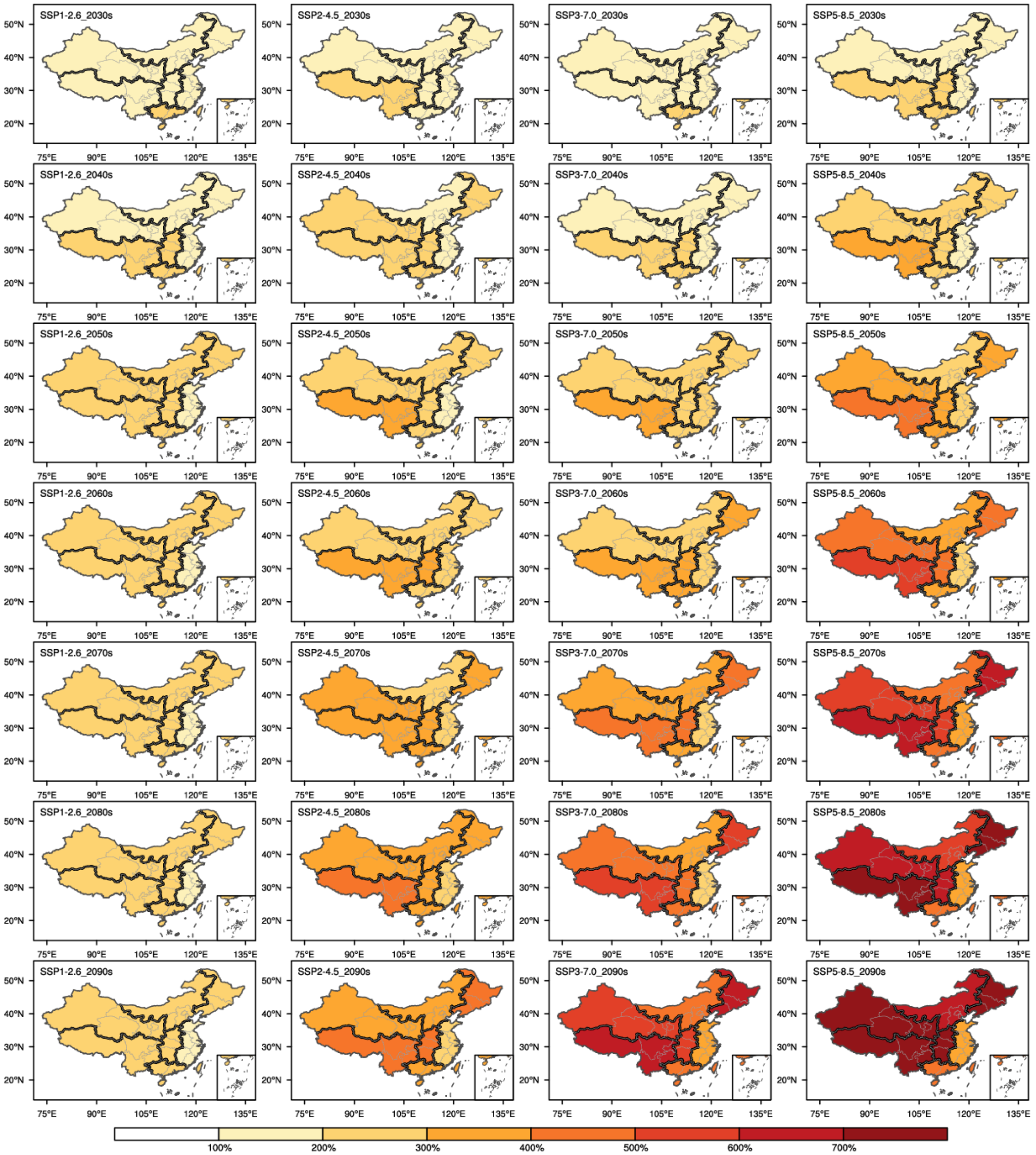

Supplementary Figure 3. Changing ratios of future heat-related mortality versus the current level averaged for every 10-year interval over 2031-2100 under SSP1-2.6, SSP2-4.5, SSP3-7.0, and SSP5-8.5.

Supplementary Table 1. Summary of the mortality for the 195 locations during the present day.

Province	City	Locations (*n*)	Non-accident Mortality (‰)
Anhui	Anqing	1	5.64
Anhui	Chaohu	1	6.86
Anhui	Hefei	8	3.99
Anhui	Maanshan	2	5.08
Anhui	Chuzhou	2	6.06
Anhui	Huainan	1	4.80
Anhui	Lu`an	4	6.66
Anhui	Wuhu	1	6.86
Beijing	Beijing	16	3.94
Gansu	Baiyin	1	4.56
Gansu	Dingxi	2	6.07
Gansu	Lanzhou	7	4.35
Gansu	Linxia	5	5.95
Hebei	Baoding	1	6.26
Hebei	Shijiazhuang	21	5.87
Heilongjiang	Daqing	1	4.19
Heilongjiang	Harbin	12	4.73
Heilongjiang	Suihua	2	5.82
Hubei	Jinmen	1	5.15
Hubei	Wuhan	1	4.29
Jilin	Jilin	4	4.29
Jilin	Liaoyuan	2	5.55
Jilin	Siping	3	4.89
Jilin	Changchun	10	4.95
Jiangsu	Changzhou	2	5.26
Jiangsu	Nanjing	11	4.58
Jiangsu	Yangzhou	3	7.46
Jiangsu	Zhenjiang	3	6.18
Liaoning	Fushun	2	6.6
Liaoning	Liaoyang	6	5.54
Liaoning	Shenyang	13	6.37
Liaoning	Tieling	1	6.25
Qinghai	Haidong	1	5.92
Qinghai	Xining	1	4.29
Shanxi	Xi'an	1	4.28
Shanxi	Yulin	1	5.64
Shanghai	Shanghai	16	4.96
Sichuan	Chengdu	16	4.83

Sichuan	Deyang	1	7.15
Sichuan	Meishan	3	7.52
Tianjin	Tianjin	1	4.14
Xinjiang	Urumqi	1	2.46
Yunnan	Kunming	1	6.1
Zhejiang	Hangzhou	1	4.83
Guangdong	Guangzhou	1	3.63

Supplementary Table 2. Basic information about the climate model simulations used in this study

**Models**	**Institution**	**Country/Region**	**Member**
ACCESS-CM2	CSIRO	Australia	r1i1p1f1
ACCESS-ESM1-5	CSIRO	Australia	r1i1p1f1
CanESM5	CCCma	Canada	r1i1p1f1
CESM	NCAR	USA	r4i1p1f1
CMCC-CM2-SR5	CMCC	Italy	r1i1p1f1
CMCC-ESM2	CMCC	Italy	r1i1p1f1
CNRM-CM6-1	CNRM-CERFACS	France	r1i1p1f2
CNRM-ESM2-1	CNRM-CERFACS	France	r1i1p1f1
EC-Earth3	EC-Earth-Consortium	Europe	r1i1p1f1
EC-Earth3-Veg-LR	EC-Earth-Consortium	Europe	r1i1p1f1
FGOALS-g3	CAS	China	r1i1p1f1
GFDL-ESM4	MOHC	UK	r1i1p1f1
INM-CM4-8	INM	Russia	r1i1p1f1
INM-CM4-8	INM	Russia	r1i1p1f1
IPSL-CM6A-LR	IPSL	France	r1i1p1f1
KACE-1-0-G	NIMS-KMA	South Korea	r1i1p1f1
MIROC-ES2L	MIROC	Japan	r1i1p1f2
MIROC6	MIROC	Japan	r1i1p1f1
MPI-ESM1-2-HR	MPI	Germany	r1i1p1f1
MPI-ESM1-2-LR	MPI	Germany	r1i1p1f1
MRI-ESM2-0	MRI	Japan	r1i1p1f1
NorESM2-LM	NCC	Norway	r1i1p1f1
NorESM2-MM	NCC	Norway	r1i1p1f1
TaiESM1	AS-RCEC	China	r1i1p1f1
UKESM1-0-LL	MOHC	UK	r1i1p1f2

Supplementary Table 3. Annual heat-related mortality in China and the subregions for present day

	**Heat-related mortality (95% CI)**
**China**	0.045‰ (0.031-0.058‰)
**NE**	0.020‰ (0.007-0.032‰)
**NC**	0.054‰ (0.032-0.075‰)
**NW**	0.016‰ (0.009-0.024‰)
**EC**	0.067‰ (0.053-0.081‰)
**CC**	0.032‰ (0.018-0.046‰)
**SW**	0.039‰ (0.022-0.055‰)
**SC**	0.096‰ (0.069-0.122‰)
